# The role of thoracic epidural anesthesia in severe acute pancreatitis

**DOI:** 10.1186/cc13718

**Published:** 2014-02-07

**Authors:** Daniel Harper, Clare E McNaught

**Affiliations:** 1 Combined Gastroenterology Research Group, Scarborough Hospital, Woodlands Drive, Scarborough YO16 6QL, UK

## Abstract

In animal studies of severe acute pancreatitis, thoracic epidural anesthesia appears to enhance the splanchnic circulation, improve end-organ perfusion, and favorably influence mortality. The application of thoracic epidurals in the critically ill human patient is less clear. Methodological difficulties in reliably assessing mesenteric flow have hampered progress, and clinical concerns surrounding this potentially attractive therapeutic modality remain unanswered. Future research needs to focus on the impact of epidural anesthesia on basic human physiological pmeters to help direct further randomized studies in human disease.

## 

In recent years, researchers have examined a number of therapeutic modalities aimed at maintaining the splanchnic circulation, in the hope of modifying the host cytokine response to the insult causing pancreatitis. In the previous issue of *Critical Care*, Bachmann and colleagues [1] explored the hypothesis that regional sympathetic blockade, through the use of thoracic epidural anesthesia (TEA), may promote splanchnic flow and improve pancreatic oxygenation in a porcine model of severe acute pancreatitis (SAP). Acute pancreatitis is a common surgical emergency with an annual case incidence of 15 to 35 per 100,000 population. The recently published Revised Atlanta Classification has stratified the disorder into mild, moderate, and severe categories. SAP is associated with a mortality rate of up to 30% and is characterized by the persistence of a systemic inflammatory response 48 hours after the onset of the attack [2]. The pathophysiology of severe pancreatitis is yet to be fully elucidated, but inadequate pancreatic microvascular perfusion and hypoxia may play a significant role in early disease progression [3].

In Bachmann and colleagues’ well-designed study, severe pancreatitis was induced in 34 anesthetized pigs through an intraductal infusion of bile acid followed by ligation of the pancreatic duct. Animals were randomly assigned to receive SAP alone or SAP with a TEA infusion started 75 minutes after SAP was initiated. Over the course of a 6-hour period, pancreatic tissue oxygen tension and microcirculation were directly measured by using a polarographic probe and laser Doppler imager, respectively, after which anesthesia was ceased and the animals were closely monitored for 7 days prior to sacrifice. Reductions in tissue oxygenation and microcirculation were observed after induction of SAP in both groups, but perfusion and oxygenation significantly improved when TEA was started in the treatment group. In addition, survival was significantly greater in the TEA group than the control, which had mortality rates of 29.4% and 82.4%, respectively.

This study provides further support to the theory that changes in pancreatic microcirculation and tissue oxygenation contribute to the progression of SAP [4]. The beneficial effect of TEA in this study appeared to relate specifically to the effects of sympathetic blockade on the splanchnic circulation and not overall perfusion, as global indices of circulation such as cardiac output and blood pressure were tightly controlled. Standardization of intravascular volume replacement in each group was critical, as previous studies have shown that different intravenous fluid preptions can independently influence the outcome of SAP [5]. The position of the epidural catheter (T7/8) was confirmed by epidurogram both at the start of the procedure and after sacrifice. Definitive proof of epidural spread was not established, and so the extent of the sympathetic block and the involvement of the nervi accelerantes could not be assessed.

These results are undoubtedly interesting, but how applicable are they to our daily clinical practice? At present, controversy still exists as to the effects of thoracic epidurals on the human splanchnic circulation. Some clinical trials, in which mesenteric blood flow was recorded directly, demonstrated a reduction in intestinal perfusion, which was not corrected with the administration of intravenous fluids alone. Conversely, other human studies demonstrated increases in hepatic blood flow and gastric mucosal blood flow by using surrogate markers of tissue perfusion. In animal models of shock and sepsis, TEA seems to have a protective effect in preventing splanchnic vasoconstriction, but the physiological response may be different in humans. Furthermore, research in humans has been hampered by the lack of a robust methodological technique to measure splanchnic flow non-invasively [6].

The use of TEA in patients with SAP poses further clinical problems. At what point in the disease course should the epidural be placed? What about the risk of coagulopathy and epidural hematoma, particularly in patients with a biliary etiology who may well be jaundiced? How long do you continue TEA, when the recovery from an episode of SAP can take weeks and there is evidence that infective complications increase with the length of time that epidurals are *in situ*? Hematological markers of infection may be raised due to the acute inflammatory response in pancreatitis, masking any signs of epidural-related sepsis. How do you detect symptoms of increasing back pain or deteriorating motor function when the patients may have impaired cognition or require invasive ventilation? Although some initial feasibility studies of TEA in SAP have been performed, the numbers of patients assessed are too low to show any meaningful difference in the significant, but rare, complications of TEA [7]. Despite this, as the risk of significant morbidity from SAP is so high, it may be reasonable to accept a small rate of TEA complications if a major reduction in mortality was confirmed.

The use of TEA in the acute clinical setting of SAP and sepsis remains a fascinating area of research. Reliable non-invasive measures of splanchnic perfusion require development to allow further evaluation of the effect of TEA on basic human physiological pmeters and to help direct randomized trials in critically ill patients.

## Abbreviations

SAP: Severe acute pancreatitis; TEA: Thoracic epidural anesthesia.

## Competing interests

The authors declare that they have no competing interests.
